# Identification of primordial germ cell-like cells as liver metastasis initiating cells in mouse tumour models

**DOI:** 10.1038/s41421-020-0145-3

**Published:** 2020-03-24

**Authors:** Chunfang Liu, Zhan Ma, Zhen Cai, Fengyu Zhang, Cheng Liu, Tingjin Chen, Danni Peng, Xiaohong Xu, Hui-Kuan Lin

**Affiliations:** 1 Department of Laboratory Medicine, Huashan Hospital, Shanghai Medical College, Fudan University, Shanghai, 200040 China; 2 Department of Cancer Biology, Wake Forest School of Medicine, Winston-Salem, NC 27157 USA; 3 Department of breast surgery, First Affiliated Zhejiang Provincial Hospital of Traditional Chinese Medicine, Hangzhou, 310006 China

**Keywords:** Cancer, Mechanisms of disease

## Abstract

Liver metastasis, characterized by the spread of tumors to the liver from other areas, represents a deadly disease with poor prognosis. Currently, there is no effective therapeutic strategies and/or agents to combat liver metastasis primarily due to the insufficient understanding of liver metastasis. To develop a promising strategy for targeting liver metastasis, understanding of a cell origin responsible for liver metastasis and how this cell can be pharmacologically eliminated are therefore crucial. Using diverse tumor models including *p53*^*−/−*^ genetic mouse model and syngeneic tumor models, we identified primordial germ cell (PGC)-like tumor cells, which are enriched in earliest liver micro-metastasis (up to 99%), as a cell origin of liver metastasis. PGC-like tumor cells formed earliest micro-metastasis in liver and gradually differentiated into non-PGC-like tumor cells to constitute late macro-metastasis in the course of tumor metastasis. The liver metastasis-initiating cells (PGC-like tumor cells) display cell renewal and differentiation capabilities, resemble primordial germ cells (PGCs) in morphology and PGC marker gene expression, and express higher level of the genes linked to metastasis and immune escape compared with non-PGC-like tumor cells. Of note, Stellar^high^ PGC-like tumor cells, but not Stellar^low^ non-PGC-like cells, sorted from primary tumors of *p53*^*−/−*^ mice readily form liver metastasis. Depletion of PGC-like tumor cells through genetic depletion of any of key germ cell genes impairs liver metastasis, while increased PGC-like tumor cells by SMAD2 knockout is correlated with markedly enhanced liver metastasis. Finally, we present the proof of principle evidence that pharmacologically targeting BMP pathways serves as a promising strategy to eliminate PGC-like tumor cells leading to abrogating liver metastasis. Collectively, our study identifies PGC-like tumor cells as a cell origin of liver metastasis, whose depletion by genetically targeting core PGC developmental genes or pharmacologically inhibiting BMP pathways serves a promising strategy for targeting liver metastasis.

Metastasis is a complex process that involves multiple steps of biological events. It determines malignant aggressiveness and often causes drug resistance, thereby accounting for the major cause of tumor-associated death^1,2^. Although the substantial efforts in the last several decades have been devoted to understanding the factors and/or genetic programs regulating cancer metastasis^1,3–5^, it remains unclear what type of tumor cells could initiate cancer liver metastasis. Clinically, metastasis is a remarkably inefficient process due to the fact that only a small cell population from bulk tumor cells could drive metastatic initiation^1,2^. Thus, identification of the seed for cancer metastasis would not only provide better understanding of cancer metastasis, but also offer a new and effective strategy to target cancer metastasis. In this study, we aimed to identify a tumor cell origin for liver metastasis using diverse mouse tumor models and developed a pharmacological strategy to eradicate this tumor cell origin for targeting liver metastasis.

## PGC-like cells appear in early liver metastases using diverse tumor models

**Fig. 1:**
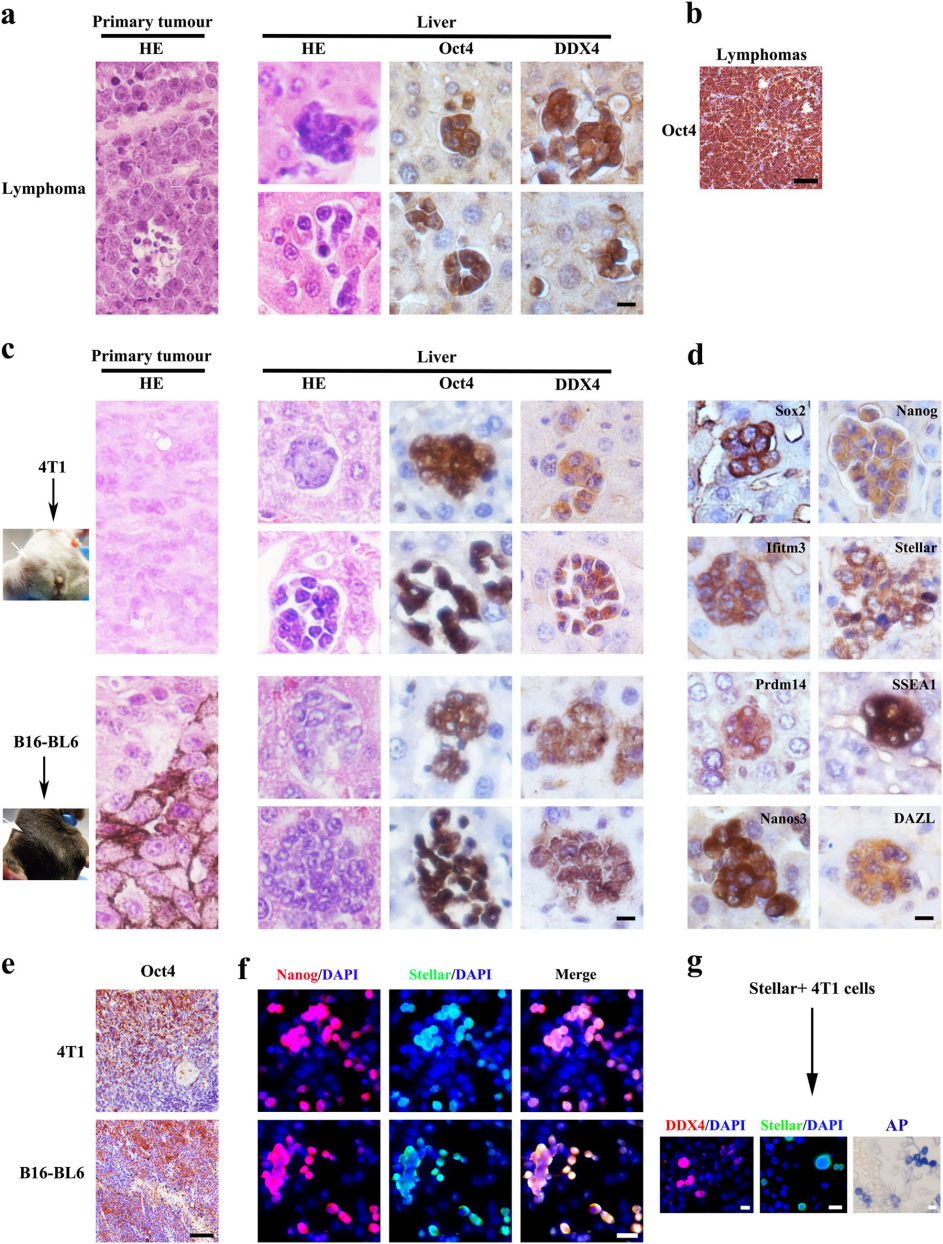
PGC-like subpopulation appears in early liver metastasis. **a** Primary lymphoma sections and liver tissues derived from *p53*^−*/*−^ mice were stained with H&E or primary antibody against indicated proteins. **b** Lymphoma sections from *p53*^−*/*−^ mice were stained with Oct4 antibody. **c** 4T1 breast cancer cells were injected into mammary fat pad of BALB/c mice, while B16-BL6 melanoma cells were injected subcutaneously into C57BL/6 mice. 4T1 and B16-BL6 primary tumors and their corresponding liver tissues were stained with H&E or the primary antibody against the indicated protein. **d** Representative sections of liver metastasis from 4T1 or B16-BL6 subcutaneous primary tumor were stained with the primary antibody against the indicated protein. **e** Primary Tumor sections from 4T1 and B16-BL6 cells were stained with antibody against indicated proteins. **f** The cultured 4T1 and B16-BL6 cells were stained with primary antibody against the indicated protein. **g** The fluorescent image of the daughter cells derived from Stellar^high^ 4T1 cells 3 weeks after cultured. Scale bar = 20 μm in (**a**, **c**, **d**, **g**), 50 μm in (**b**, **e**, **f**).

Loss of p53 tumor suppressor is one of the most frequent events found in advanced human cancers^3,6,7^. p53 inactivation not only promotes cancer initiation, but also cancer progression and metastasis^3^. p53 deficient mice develop spontaneous lymphoma with highest incidence, followed by sarcoma^8,9^. While p53 loss has been clearly linked to the progression of cancer metastasis, it has not been reported that p53 deficient mice develop overt metastasis, likely due to the fact that p53 deficient mice die of cancer in a fairly quick fashion, which does not offer sufficient time for metastasis development. However, by carefully examining hematoxylin and eosin (HE) staining of hepatic tissues from *p53*^*−/−*^ mice, we surprisingly observed hepatic micro-metastasis in all those *p53*^*−/−*^ mice that have developed lymphoma (12/12), but not in wild-type (WT) mice. We found that this subpopulation of tumor cells was about 7–12 μm in diameter, round-oval in shape and displayed a large and spherical nucleus with a high ratio of nucleus to cytoplasm (Fig. 1a). The majority of these cells formed cell spheres with high cell–cell contact, although a few of them were individual and separated from each other (Fig. 1a). It seemed that most of these cells appeared in hepatic sinusoid and exhibited an undifferentiated state in morphology, likely representing liver metastasis-initiating cells. Notably, the uniquely morphological features presented in this cell population are reminiscent of PGCs, which similarly display a round-oval shape in morphology with a high ratio of nucleus to cytoplasm, often cluster together and exhibit tight cell–cell contact in the early phase of migration process, but physically separate from each once arriving to the gentile ridge^10,11^. To determine whether this small subset of tumor cells are indeed PGC-like tumor cells, we examined the expression of germ cell-specific markers^10^, including Oct4 (one of the earliest markers) and DDX4 (a marker of post-migratory germ cells) at the different developmental stages. Similar to the PGCs, this tumor cell population was positive for Oct4 and DDX4 (Fig. 1a), indicative of PGC-like cells. The similar PGC-like subpopulation of tumor cells was also detected in primary lymphomas in *p53*^*−/−*^ mice^9^ (Fig. 1b). Thus, our data indicate that PGC-like cells observed in liver micro-metastasis in *p53*^*−/−*^ mice likely derive from primary thymic lymphomas.

To validate the finding that PGC-like tumor cells are detected in liver metastasis, we used two additional syngeneic tumor models that develop spontaneous liver metastasis by injecting 4T1 and B16-BL6 tumor cells into BALB/c mice and C57BL/6 mice (subcutaneous injection), respectively. Notably, the similar PGC-like subpopulation of tumor cells expressing both Oct4 and DDX4 was also observed in liver micro-metastasis derived from both 4T1 and B16-BL6 tumors (Fig. 1c). Moreover, a series of other germ cell-specific markers, including Nanog, SSEA1, Sox2, Prdm14, Stellar, Ifitm3, Nanos3, and DAZL were highly expressed in PGC-like tumor cells (Fig. 1d), but rarely detected in the full differentiated somatic cell-like subpopulation (Supplementary Fig. S1a), further validating their similarity to natural PGCs^10^.

The similar PGC-like subpopulation of tumor cells was also detected in primary tumor (Fig. 1e) and in vitro cell cultures (Fig. 1f) derived from 4T1 and B16-BL6 cells. To know whether PGC-like tumor cells have the ability to self-renew and differentiate into non-PGC-somatic cells, we isolated Stellar^high^ tumor cells (Stellar is a germ cell-specific marker)^10,11^ as surrogates for PGC-like cells from 4T1 tumor cells for cell differentiation assay under in vitro cell culture. The Stellar^high^ cells could attach to the dish and grew (Fig. 1g). Three weeks after culture, Stellar^high^ cells could differentiate into fibroblast-like cells likely representing somatic tumor cells, while maintaining a small subset of germ cell-like populations (Fig. 1g). Thus, PGC-like tumor cells have self-renewal and differentiation capability. Together, the findings suggest that the occurrence of PGC-like tumor cells is common in liver micro-metastasis from multiple tumor models.

## PGC-like tumor cells as seeds for liver metastasis

**Fig. 2:**
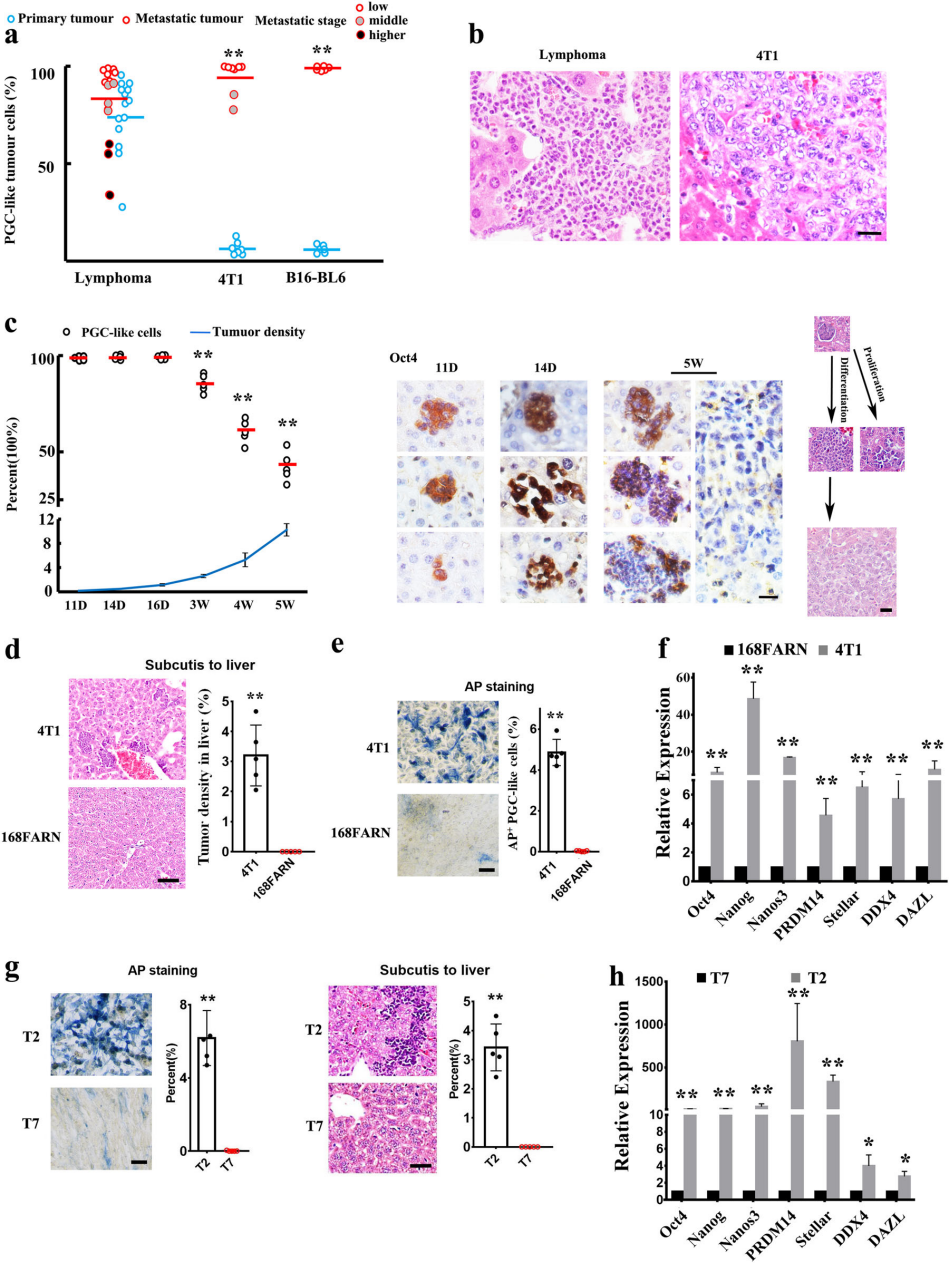
PGC-like tumor cells serve as a seed for liver metastasis. **a** The percentage of PGC-like cells in paired primary tumors and cancer liver metastasis from spontaneous lymphomas in *p53*^−*/*−^ mice, 4T1 tumors and B16-BL6 tumors. **b** The bigger cluster of tumor cells in liver tissues of the mice subcutaneously inoculated with lymphoma tumor cells in *p53*^−*/*−^ mice or 4T1 cells mainly shows the differentiated tumor cells. **c** 4T1 cells were subcutaneously injected into BALB/c mice, and the mice were sacrificed to harvest for liver tissues for histological analysis at different time points. The percentage of PGC-like cells and tumor density in the paired liver metastasis at different time points. Liver section at different time points were stained with Oct4 antibody. **d** Histological analysis of liver metastasis in mice after subcutaneously injected with 4T1 and 168FARN cells, respectively. The plot shows the percentage of liver metastasis in mice after subcutaneously injected with 4T1 and 168FARN cells, respectively. **e** Bright field image of AP staining in 4T1 and 168FARN. The plot shows the percentage of AP-positive cells in 4T1 and 168FARN cultures. **f** The real-time PCR analysis of the expression of PGC-related genes in 4T1 and 168FARN cells. **g** The percentage of AP-positive cells in TBMDCs-2 (T2) and TBMDCs-7 (T7) cultures (left panel). The percentage of liver metastasis in mice after subcutaneously injected with TBMDCs-2 (T2) and TBMDCs-7 (T7) cells, respectively (left panel). **h** The real-time PCR analysis of the expression of PGC-related genes in TMDCs-2 (T2) and TMDCs-7 (T7). **P < 0.01. Scale bar = 50 μm. Scale bar = 20 μm in (**b**, **c**), 50 μm in (**d**, **e**, **g**).

Compared with their primary tumors, the PGC-like subpopulation was markedly enriched in early liver micro-metastasis from 4T1 and B16-BL6 (Fig. 2a), indicating that PGC-like cells tend to migration. Interestingly, PGC-like tumor cells were often enriched in the early metastatic lesion where non-PGC-like somatic tumor cells were barely detected (Fig. 2a). In contrast, we found the ratio of PGC-like cells declined at later stage of metastatic cluster (Fig. 2a, b), accompanied by the appearance of non-PGC-like somatic tumor cells, as determined by loss of the expression of germ cell-specific markers (Supplementary Fig. S1a), indicating that somatic non-PGC-like cells likely differentiated from PGC-like tumor cells during the metastasis propagation. Compared with B16-BL6 cells, 4T1 cells exhibited a better ability to confer liver metastasis, although B16-BL6 tumors grew faster than 4T1 tumors. It should be noted that liver metastases from B16-BL6 cells at different time points remained still at early stage, and virtually all metastatic cell clusters (~99%) represented PGC-like cells (Fig. 2a). These findings raise the possibility that the PGC-like tumor cells may serves as seeds for liver metastasis.

To further validate the potential role of PGC-like tumor cells in the metastatic process, we sought to monitor liver metastasis derived from 4T1 tumors at various time points. At early stage, the majority of tumor cells were PGC-like tumor cells (up to 99%) with very few somatic tumor cells observed in liver micro-metastasis (Fig. 2c). With the progression of metastasis, the ratio of PGC-like tumor cells gradually declined, accompanied by the increased ratio of somatic tumor cell cluster (Fig. 2c and Supplementary Fig. S1b–c). Moreover, we observed that PGC-like cells could propagate and differentiate into somatic tumor cells leading to larger metastatic cluster during metastatic progression (Fig. 2c), correlated well with Oct4 expression. The Oct4 protein was highly expressed in small metastatic cluster at early stage, but gradually declined in larger metastatic clusters and eventually undetected in well-differentiated metastatic tumor cells at later stage (Fig. 2c). Thus, the change of the Oct4 expression could monitor the early expansion of metastatic tumor cells. Together, these findings indicate that the PGC-like cells may serve as seeds for the initiation of liver metastasis.

To further define the role of PGC-like tumor cells in liver metastasis, we used the isogenic cell subpopulations including metastatic (4T1) and non-metastatic (168FARN) tumor cells^12^ derived from the same mouse mammary tumor for tumourigenesis and metastasis assays. Consistent with the previous study^12^, while both 4T1 and 168FARN cells subcutaneously injected to the mice formed primary tumor, 4T1, but not 168FARN, cells developed liver metastasis (Fig. 2d). The difference in metastasis potential between these cell lines was sharply correlated with the number of PGC-like tumor cells present in these cell lines, as determined by non-specific alkaline phosphatase (AP) staining, which is used for the identification of PGCs since AP is a maker for PGCs^10^ (Fig. 2e) and other PGC marker gene expression (Fig. 2f), indicating that PGC-like cells may play a crucial role in liver metastasis. The similar results with positive correlation between the number of PGC-like tumor cells and liver metastasis were also observed in the transformed bone marrow-derived cells-7 (TBMDCs-7, p53wt) and TBMDCs-2 (*p53*^*−/−*^) (Fig. 2g, h). Together, these findings suggest that the PGC-like tumor cells may serve as seeds for liver metastasis.

## A crucial role of PGC-like tumor cells in hepatic colonization of tumor cells

**Fig. 3:**
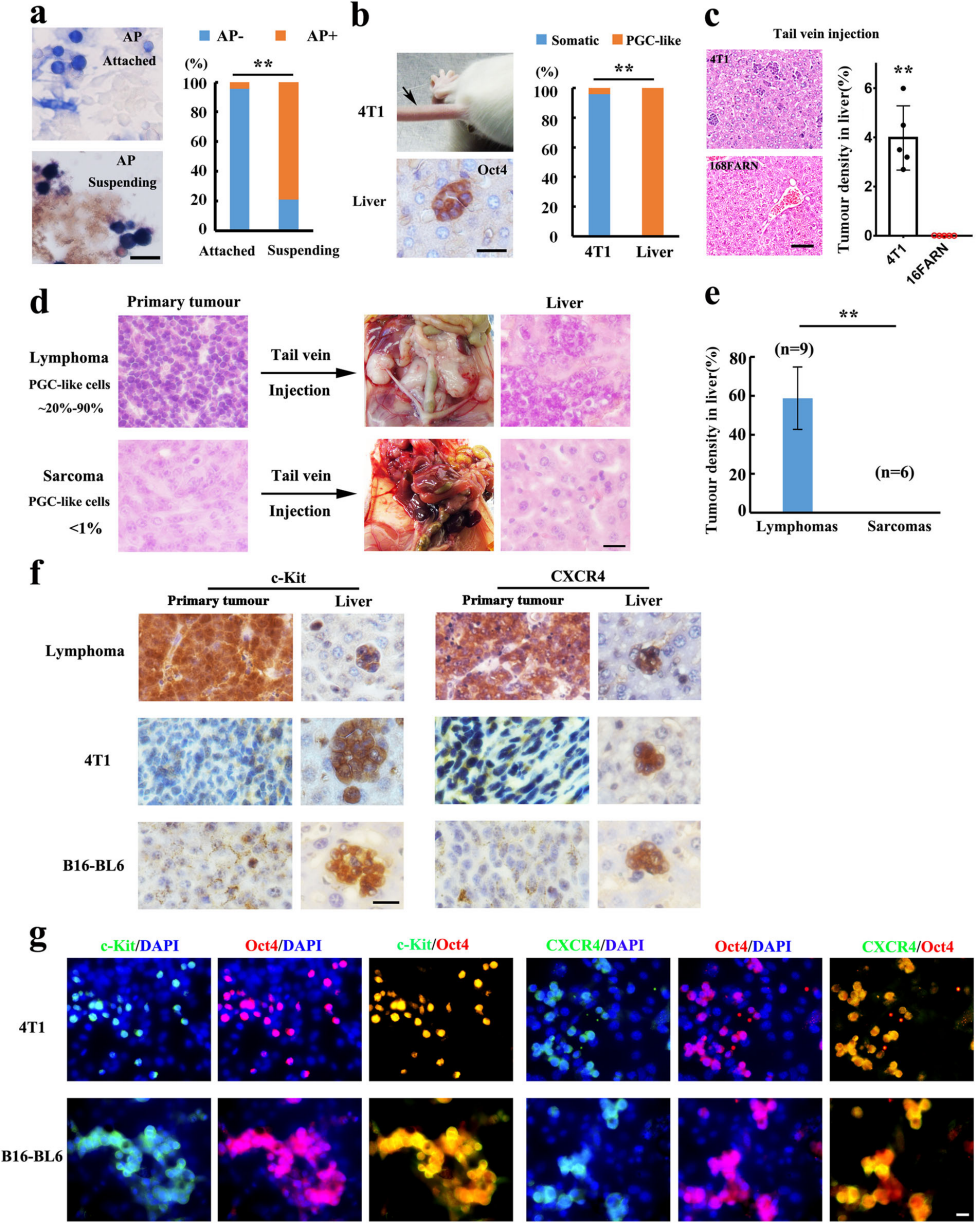
PGC-like cells display the similar characteristics of natural PGCs. **a** Images of the PGC-like tumor cells with AP staining in adherent and suspended 4T1 cells. The plots show the percentage of PGC-like cells with AP staining in adherent and suspended 4T1 cells. **b** The liver tissue from the mice inoculated with 4T1 cells through tail vein was stained with Oct4 antibody. The percentage of PGC-like subpopulation and somatic tumor subpopulation detected in liver was shown. **c** Colonization to liver in mice inoculated with 4T1 cells and 168FARN 15 day after the tail vein injection. Bright field image of liver colonization (left plane). The plot shows the percentage of tumor cell colonization to liver (right plane). **d** Lymphoma or sarcoma tumor cells of *p53*^−*/*−^ mice were injected into nude mice through tail vein. The sections of metastatic hepatic tissues were stained with H&E 3 weeks after injection. **e** The percentage of metastatic tumor cells in liver of mice inoculated with lymphoma and sarcoma tumor cells of *p53*^−*/*−^ mice 3 weeks after the tail vein injection. **f** Primary tumors and liver tissues of the mice subcutaneously injected with various tumor cells were stained with the antibody for the indicated protein. **g** Cultures were stained with the primary antibody against the indicated protein. **P < 0.01. Scale bar = 20 μm (**a**, **b**, **g**), 50 μm (**d**, **f**), 100 μm (**c**).

For full-blown metastasis, metastasis-initiating cells require to gain the capability to migrate and survive in the circulation and distant sites composed of diverse stresses and immune cells that could potentially kill tumor cells^1,2,4^. Therefore, we firstly investigated the migration potential of PGC-like tumor cells in 4T1 cell cultures. The AP staining, which is generally used for defining PGC cells^10^, showed that about 80% of suspended survival cells was PGC-like tumor cells (Fig. 3a), although the ratio of PGC-like tumor cells was only about 4% in cultured 4T1 cells (Fig. 3a), indicative of the motile nature of PGC-like cells^10,13^ (Fig. 3a). Importantly, these suspended 4T1 cells could give rise to tumor in vivo when injected into nude mice with only 100 cells (Supplementary Fig. S2a).

Clinical studies showed that only a small subset of circulating tumor cells can confer metastatic colonies, suggesting that the main barrier for metastasis likely occurs during the colonization of tumor cells to the secondary site^1,2^. To understand which subset of tumor cells could successfully colonize to the secondary site, we inoculated 4T1 cells into BALB/c mice through the tail vein to investigate the colonization and survival of various subpopulations in hepatic tissues. Six days after injection, PGC-like tumor cells, but not somatic tumor cells, were detected in liver micro-metastasis, although PGC-like tumor cell population versus somatic tumor cell population is about 4% versus 96% in those injected 4T1 cells (Fig. 3b), suggestive of the crucial role of PGC-like subpopulation in hepatic colonization. Consistently, 168FARN cells with few PGC-like cells failed to colonize and form liver metastasis in mice upon tail vein injection compared with 4T1 cells (Fig. 3c).

Although sarcomas and lymphomas were developed in *p53*^*−/−*^ mice with the similar genetic background, the percentage of PGC-like tumor cells was far higher in lymphomas (ranging from 20 to 90%) (Fig. 1b) than sarcomas (<1%)^9^ (Supplementary Fig. S2b). To further consolidate distinct colonization potential in liver between somatic tumor cells and PGC-like tumor cells, we inoculated tumor cells from sarcomas or lymphomas of *p53*^*−/−*^ mice into nude mice through tail vein injection for the metastasis assay. Notably, tumor cells from lymphomas could colonize to various tissues, but not lung, including liver, kidney and ovary within 3 weeks (Fig. 3d, e and Supplementary Fig. S2c), indicating that the PGC-like subpopulation from lymphomas displays a unique propensity to colonize to distinct organs. The difference possibly attributes to the distinct niche environments between liver and lung, which selectively allow for low PGC-like cells and high PGC-like cells to colonize and survive, respectively^1,2^. On the contrary, tumor cells from sarcomas failed to colonize to the ovary, kidney and liver, but instead colonized to lung (Fig. 3d, e and Supplementary Fig. S2c). Thus, lymphoma tumor cells with high percentage of PGC-like tumor cells prefer to colonize to the tissues such as liver, while the sarcoma tumor cells with low percentage of PGC-like cells display a higher propensity to lung^1,2,4^. These data further suggest that PGC-like tumor cells may play a critical role in driving liver metastasis.

## PGC-like tumor cells express several markers related to migration and immune escape

As PGC-like cell population displays stronger migration, colonization and survival in liver than non-PGC-like cell population, we determined whether there is any connection of certain unique proteins to these phenotypes with respect to metastasis. To that end, we sought to examine the expression of those proteins critically involved in migration, colonization and survival in PGC-like cells versus non-PGC-like cells by immunohistochemistry staining. Of note, we observed that CXCR4 and c-Kit, two crucial factors in cell migration and colonization of PGCs^14–16^ and tumor^17,18^, were highly expressed in PGC-like cells within liver micro-metastasis, but rarely detected in somatic differentiated clusters (Fig. 3f), correlated with the stronger metastatic potential of the PGC-like subpopulation. We found that CXCR4 and c-Kit also co-expressed with Oct4 indicative of PGC-like cells^14–16^, but they were almost undetectable in Oct4^–/low^ somatic tumor cells under in vitro cell culture of various cell lines (Fig. 3g). These data offer the possibility that CXCR4 and c-Kit, upregulated and correlated with various tumor metastases, may be used to define PGC-like subpopulation of tumor cells in metastatic cancers^17,18^.

**Fig. 4:**
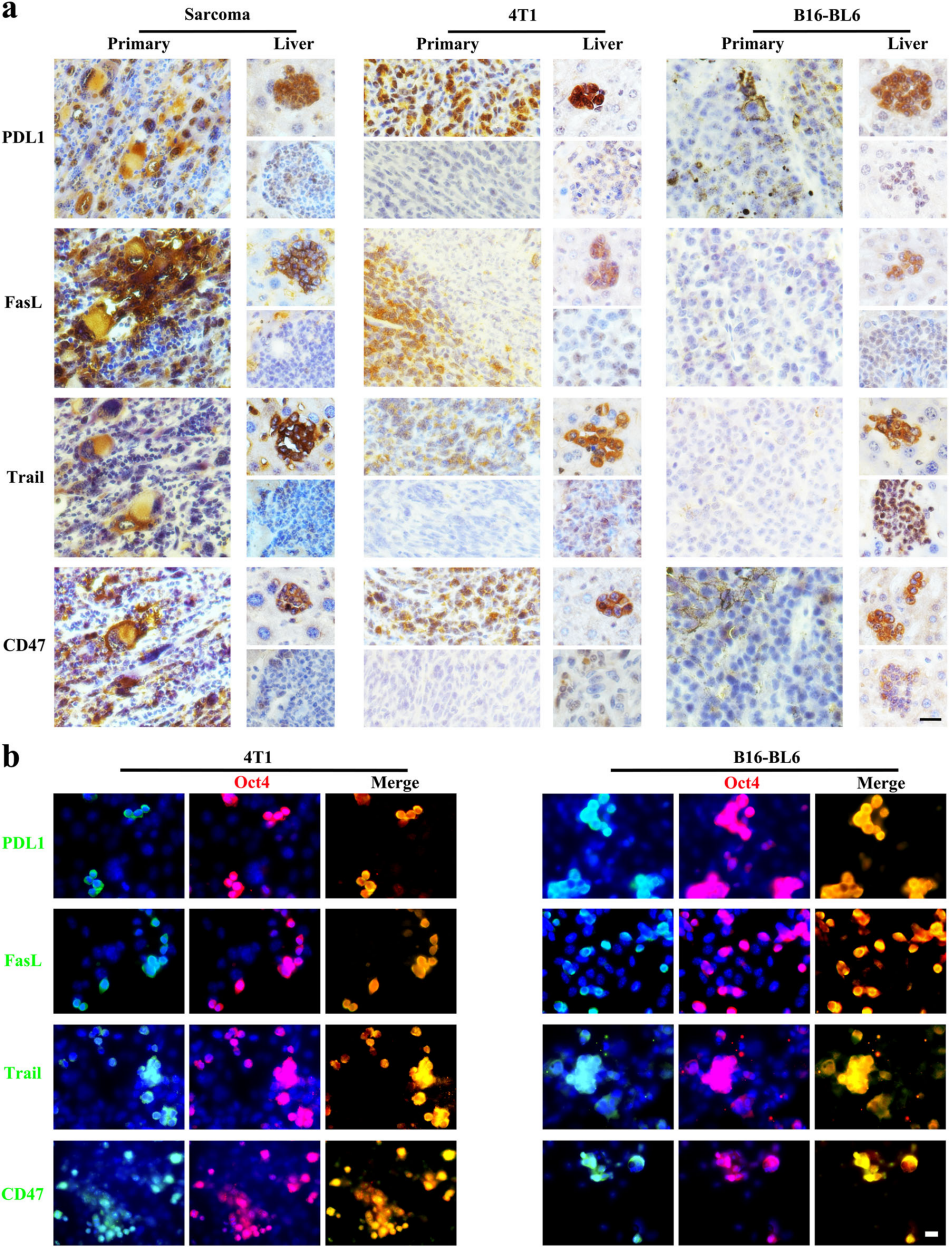
Expression of markers related with immune escape in PGC-like tumor cells. **a** Primary tumors and liver tissues of the mice subcutaneously injected with various tumor cells were stained with the antibody for the indicated protein. **b** 4T1 and B16-BL6 cultures were stained with the primary antibody against the indicated protein. Scale bar = 20 μm (**b**), 50 μm (**a**).

Our finding that the appearance of PGC-like tumor cells in early liver metastasis in mice with intact immune system indicates that PGC-like tumor cells are capable of overcoming the physical and biological hurdles as well as immune surveillance (Supplementary Fig. S3a). Successful colonization and metastasis of tumor cells to distinct organs requires their ability to escape from immune cell attack. As PDL1, FASL, TRAIL, and CD47^19,20^ are well known to play an important role in mediating immune suppression, we determined whether germ cell-like tumors display the signature of immune suppressive genes. Notably, germ cell-like tumor cells showed stronger staining for PDL1, FASL, TRAIL, and CD47 compared with differentiated somatic tumor cells both in primary tumors and liver metastases (Fig. 4a). Consistently, PDL1, FASL, TRAIL, and CD47 also co-expressed with Oct4, but were barely detected in somatic tumor cells under in vitro cell culture (Fig. 4b). Thus, the expression of PDL1, FASL, TRAIL, and CD47^19,21^ may endow PGC-like tumor cells with the capability to escape from immune cell attack. Taken together, the expression of these markers may explain why PGC-like tumor cells could serve as a potential cell origin for tumor cell colonization and survival in a distant site, leading to liver metastasis.

## Increase of PGC-like cell formation by SMAD2 deletion is correlated with enhanced liver metastasis

**Fig. 5:**
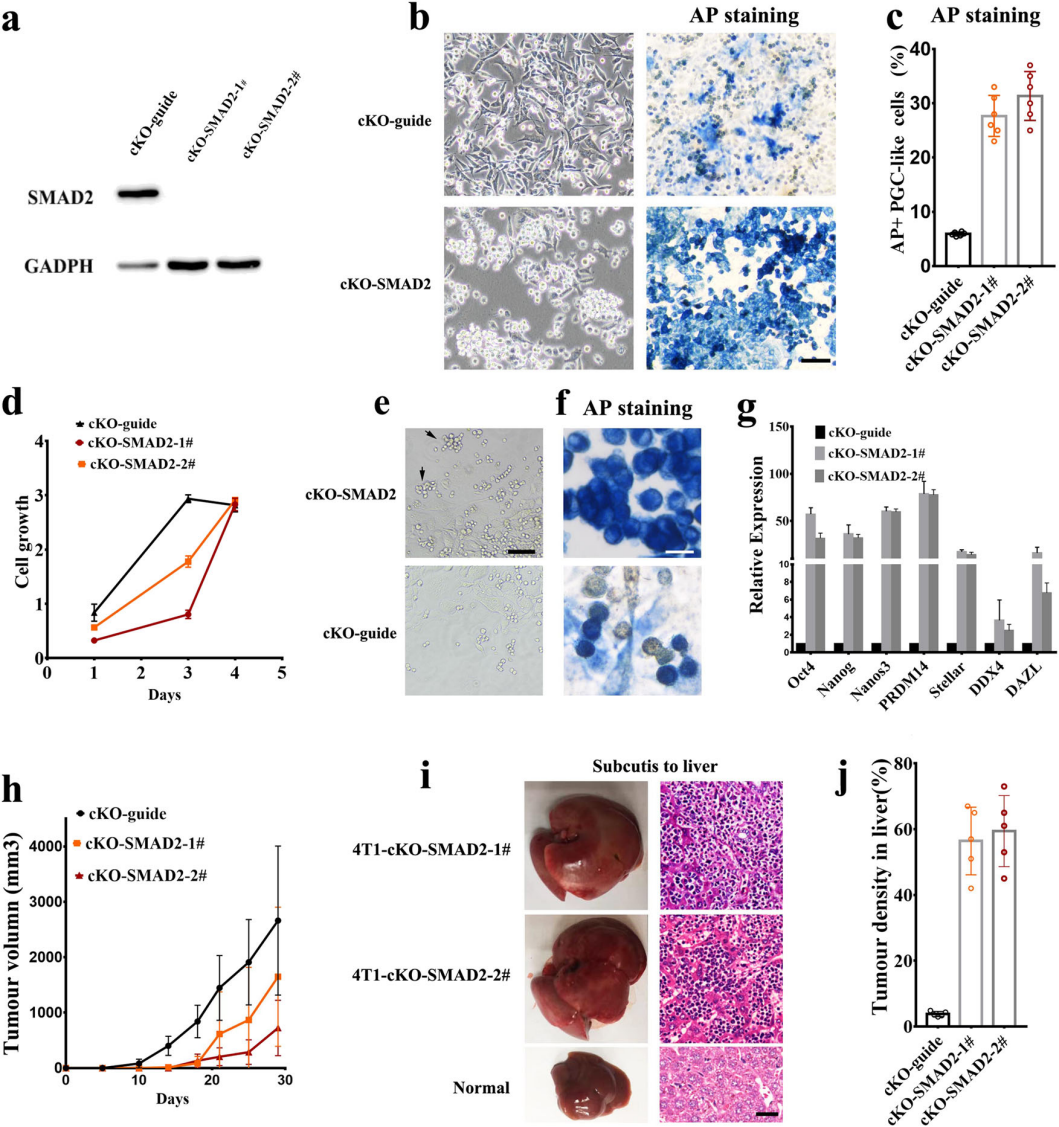
Knockout of SMAD2 promotes PGC-like cell formation and liver metastasis. **a** The SMAD2 immunoblotting was shown in 4T1 cells with control and SMAD2 knockout using CRISPR–Cas9 technology. **b** The image of 4T1 with control and SMAD2 knockout in cell growth and AP staining. **c** The percentage of AP^+^ cells in 4T1 with control and SMAD2 knockout. **d** Cell growth curve of 4T1 with control and SMAD2 knockout. **e** The PGC-like clusters migrated to new place after cultured in 96-well at single cell level. **f** AP staining of the PGC-like cell clusters. **g** The real-time PCR analysis of the expression of PGC-related genes in 4T1 cells with control and SMAD2 knockout. **h** Tumor growth curve of 4T1 with control and SMAD2 knockout after subcutaneously injected into mice. **i** Image of liver and liver HE staining derived from mice subcutaneously injected with 4T1-KO-SMAD2 cells. Normal liver as a control. **j** Tumor density in liver from the mice subcutaneously injected with 4T1 cells with control and SMAD2 knockout 30 days after injection. **P < 0.01. Scale bar = 20 μm (**f**), 50 μm (i), 100 μm (**b**, **e**).

During the course of PGC specification, SMAD2 loss causes increased numbers of PGCs^10,22^. We then investigated whether increase of PGC-like tumor cells by SMAD knockout is correlated with enhanced liver metastasis by genetically depleting SMAD2 in 4T1 cells using Cas9/CRISPR editing approach (Fig. 5a). After CRISPR-KO SMAD2, the 4T1 cells showed a distinct feature of growth (Fig. 5b). Compared with control cells (4T1-KO-guide), the 4T1-KO-SMAD2 cells exhibited a slight decrease in proliferation but increased PGC-like cells and migration, especially in low-density culture (Fig. 5b–g). When 4T1-KO-SMAD2 cells were cultured at single cell level in a 96-well plate or under low-density culture, the formation of PGC-like cell spheres, departed from plate and suspended in the medium, could be easily observed, resembling motile PGCs^13^, followed by migrating and attaching to new place to generate new cell clusters (Fig. 5e, f). Notably, while 4T1-KO-SMAD2 cells subcutaneously injected into BALB/c mice displayed reduced tumor growth (Fig. 5h), they caused enlarged liver and robustly promoted liver metastasis in pathological sections (Fig. 5i, j). These data collectively suggest that SMAD2 depletion promotes PGC-like cells and liver metastasis. Since SMAD2 has widespread effects other than the increase of PGC-like cells, it is unclear whether the promoting effect on liver metastasis by SMAD2 knockout is solely from increase of PGC-like cells.

## Depletion of PGC-like cells by genetically silencing core PGC developmental genes or pharmacologically targeting BMP pathways impairs liver metastasis

**Fig. 6:**
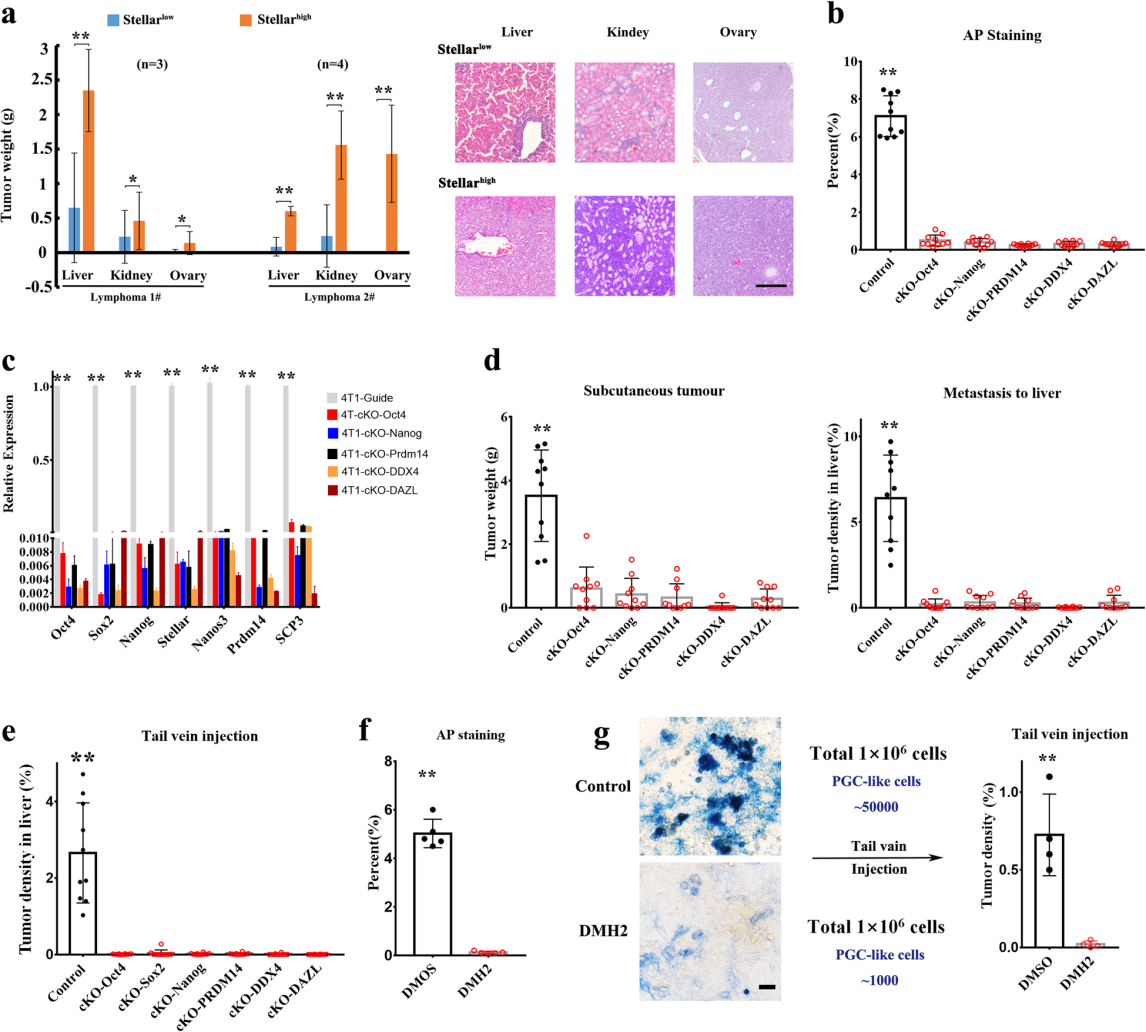
Inhibition of PGC-like cell formation by PGC developmental pathways impairs liver metastasis. **a** The tumor weight and histological analysis of various tissues from the mice inoculated with Stellar^low^ and Stellar^high^ tumor cells sorted from two different lymphomas of *p53*^−*/*−^ mice through the tail vein injection. **b** The percentage of AP-positive cells in 4T1 cells with control and knockout in diverse PGC-related genes. **c** The real-time PCR analysis of the expression of diverse PGC-related genes in control and knockout 4T1 cells. **d** The weight of primary tumor and tumor density in liver from the mice subcutaneously injected with 4T1 cells with control and knockout in diverse PGC-related genes. **e** Tumor density in liver from the mice injected with 4T1 cells with control and knockout in diverse PGC-related genes 15 days after the tail vein injection. **f** The percentage of AP^+^ cells in 4T1 cells treated with DMSO or DMH2 (6 μM). **g** Bright field image of AP staining of 4T1 cells treated with DMSO or DMH2 (6 μM). Tumor density in liver from the mice injected with 4T1 cells treated with DMSO or DMH2 for 6 days (6 μM). **P < 0.01. Scale bar = 50 μm (**g**), 200 μm (**a**).

To further validate the role of PGC-like tumor cells in hepatic colonization and metastasis, Stellar^high^ (PGC-like subpopulation) or Stellar^low^ (lymphocyte-like subpopulation) tumor cells^9,10,13^ sorted from thymic lymphomas of *p53*^*−/−*^ mice were injected into the tail vein of nude mice. Compared with Stellar^low^ cells, Stellar^high^ cells displayed much higher metastasis potential in a variety of tissues (Fig. 6a and Supplementary Fig. S4a). Notably, Stellar^high^ PGC-like cells, but not Stellar^low^ non-PGC-like cells, readily colonized to the ovary resembling the homing of natural PGCs^10^ and formed metastatic tumors (Fig. 6a and Supplementary Fig. S4a). Moreover, Stellar^high^ cells also displayed much stronger metastasis potential in liver and kidney than Stellar^low^ cells (Fig. 6a and Supplementary Fig. S4a). It should be noted that although Stellar^low^ cell population could also confer liver metastasis (Fig. 6a and Supplementary Fig. S4a), albeit to much lower extent, liver micro-metastasis was constituted primarily by Stellar^high^ PGC-like cells, likely resulting from the conversion of Stellar^low^ to Stellar^high^ cells during the colonization process. Collectively, these data suggest that PGC-like cells are seeds for cancer metastasis to multiple tissues including liver, kidney, and ovary.

We postulated that the regulatory mechanisms orchestrating PGC-like cell formation may be similar to those for natural PGC development. To directly validate this notion as well as examine the essential role of PGC-like tumor cells in liver metastasis, we used the CRISPR–Cas9 technology to knockout those genes critically involved in PGC specification and fate maintenance^10^, such as Oct4, Nanog, Prdm14, DDX4 and DAZL. Consistent with PGC development, the number of PGC-like cells were sharply reduced in 4T1 cells upon knockout of any of these PGC genes compared with control cells (4T1 cells KO guide gene as a control) (Fig. 6b, c and Supplementary Fig. S4b, e). Notably, depletion of PGC-like cells in 4T1 cells impaired both tumourigenesis and metastasis of 4T1 cells subcutaneously injected into BALB/c mice (Fig. 6d and Supplementary Fig. S4c). To further validate the role of PGC-like tumor cells in liver metastasis, we also established liver metastasis model by injecting 4T1 cells to BALB/c mice through the tail vein injection. Of note, depletion of PGC-likes cells in 4T1 cells by knockout of any of these PGC genes also abrogated liver metastasis of 4T1 cells compared with the control group (Fig. 6e and Supplementary Fig. S4d). Thus, PGC-like tumor cells resemble natural PGC in core regulatory gene network. We conclude that PGC-like tumor cells are critical for liver metastasis.

Liver metastasis remains still a huge challenge to target. As we have presented the genetic evidence to define the crucial role of PGC-like tumor cells in liver metastasis, it is of significance to identify an effective strategy to deplete PGC-like tumor cells for liver metastasis. BMP signaling has been shown to be essential for PGC development^10,11^. Given the similarity between PGC-like tumor cells and PGCs, we asked the question whether pharmacologically targeting BMP pathways serves as an effective mean to deplete PGC-like tumor cells for liver metastasis. Remarkably, targeting of BMP pathways by BMP receptor inhibitor DMH2 abrogated PGC-like tumor cell population in 4T1 cells (Fig. 6f, g and Supplementary Fig. S4f). Loss of PGC-like tumor cells by DMH2 treatment also impaired liver metastasis in 4T1 metastasis model (Fig. 6g and Supplementary Fig. S4g), similar to genetically targeting core PGC developmental genes. Hence, targeting PGC-like tumor cells by BMP receptor inhibitor represents a promising strategy for preventing and treatment of liver metastasis.

As cancer metastasis is a major cause of the cancer-related death^1,2^, identifying a true cancer cell origin for metastasis is instrumental for developing an effective strategy for managing cancer-related death. Our study reveals that PGC-like tumor cells are a cell origin for liver metastasis using genetic mouse models and syngeneic tumor models. The liver metastasis-initiating cells identified from this study resemble PGCs in morphology, express a serial of germ cell markers, readily colonize to liver and ovary, and display the similar regulatory network as PGCs^10,13^. Importantly, PGC-like tumor cells serve as seeds for the initial colonization of tumor cells to the liver and form a micro-metastasis. Subsequently, these cells differentiate into somatic tumor cells that propagate and constitute a macro-metastasis leading to full-blown metastasis. Since these cells highly express genes involved in immune escape^19,21^, we speculate that these unique gene signatures enriched in cell surface of these cells may enable them to escape from immune cell attack. The PGC-like characteristics of tumor cells^20,23^, which are induced by loss of p53 based on our earlier study^9^, might be key determinants in empowering a real seed for liver metastasis, lending a strong support of the embryonal/gametogenesis theory of tumors, which points to tumor metastasis corresponding to PGC migratory properties^24^.

Similar to the regulatory mechanisms for PGCs, we demonstrate that PGC-like tumor cells are also orchestrated by diverse germ cell markers^10^, such as Oct4, Nanog, Prdm14, DDX4, and BMP receptors. We present the data highlighting the crucial role of PGC-like tumor cells in liver metastasis. We show that increase of PGC-like cell formation by SMAD2 loss promotes liver metastasis, while genetical depletion of PGC-specific genes or pharmacological inactivation of BMP pathways markedly reduces PGC-like tumor cells leading to the impairment of liver metastasis. In line with our findings, it has been documented that some embryonic/germ cell-specific genes are upregulated in tumors^24–27^ and may play an important role in tumor malignant behaviors^20,28–30^. TGF-β signaling has been shown to act as a tumor suppressor during early stage of cancer development, but promotes metastasis in late stage of cancer progression^31^. It displays both tumor suppressive and tumor promoting signals, and the exact role it plays may largely depend on distinct cellular contexts^31^. While the explanation as to how loss of smad2 paradoxically reduces tumor growth in 4T1 cell is currently not clear, it is likely that the impairment of p53 tumor suppressive response in 4T1 cell (p53 null), which has been shown to be required for TGF-β-mediated tumor suppressive mechanism, may partly contribute to this unique phenomenon^32^. Our study therefore provides the proof of principle evidence that targeting PGC-like tumor cells is a promising strategy for liver metastasis. We propose that genetically targeting core PGC developmental genes or pharmacologically targeting BMP pathways could serve as a potential therapeutic strategy for eradicating liver metastasis.

## Materials and methods

### Cell culture

The 168FARN (obtained from Dr. Kounosuke Watabe) and 4T1 (from ATCC cell) cell lines were derived from the same breast tumors grown in BALB/c mice. *B16-BL6* melanoma cells were obtained from ATCC. Transformed bone marrow-derived cells-7 (TBMDCs-7) and TBMDCs-2 were isolated from p53wt and *p53*^*−/−*^ mice, respectively^9^, and induced malignant transformation by 3-methyl-cholanthrene (3-MCA)^23^. All the cells were cultured in high-glucose Dulbecco’s modified Eagle’s medium (DMEM, Hyclone) with 10% fetal bovine serum (FBS; Sigma) and 1% L-glutamine and were maintained at 37 °C with 5% CO_2_.

### In vivo animal experiments

The *p53*^−*/*−^ mice in C57BV6 × 129S4 background were described previously^9^. The spontaneous thymic lymphomas and sarcomas and liver tissues from *p53*^−*/*−^ mice were collected for histology analysis. For analyzing primary tumor and liver metastasis, 4T1 and B16-BL6 cancer cells (1 × 10^6^ cells) were subcutaneously injected into BALB/c mice and C57BL/6 mice, respectively. The primary tumors and liver tissues were collected for histology analysis. To analyze the tumourigenicity potential of PGC-like cells, 100 Stellar^high^ cells sorted from thymic lymphoma of *p53*^−*/*−^ mice were injected into nude mice and BALB/c subcutaneously. Five mice for each group were used. The mice were sacrificed when the tumor reached to 2 cm. The caliper was used to measure the tumor size, and the tumor volume was calculated by the equation: [mm^3^] = (length [mm]) × (width [mm])^2^ × 1/2.

To analyzing the difference in tumourigenicity and liver metastasis, 4T1 and 168FARN cells (1 × 10^6^ cells) were subcutaneously injected into BALB/c mice. The mice were sacrificed when the tumor size reaches about 2 cm after injection. TBMDCs-7 and TBMDCs-2 were subcutaneously injected into BALB/c mice. The mice were sacrificed when the tumor size reached about 2 cm after injection. The primary tumors and liver tissues were collected for histology analysis. To analyze the difference in tumourigenicity and liver metastasis, 4T1 cells (1 × 10^6^ cells) with control or knockout in diverse PGC-related genes using the Cas9/CRISPR approach were injected into BALB/c mice subcutaneously (10 mice for each group). Two independent knockout clones were used in these experiments. The mice subcutaneously injected with 4T1-cKO-Oct4, 4T1-cKO-Nanog, 4T1-cKO-Prdm14, 4T1-cKO-DDX4, 4T1-cKO-DAZL, 4T1-cKO-guide cells were sacrificed at day 25 after injection, while the mice subcutaneously injected with 4T1-cKO-SMAD2, 4T1-cKO-guide cells were sacrificed 30 days after injection. The mice were monitored for tumor formation daily before they were sacrificed. All animal experiments were performed under the Institutional Animal Care and Use Committee approval protocol.

### In vivo colonization assay

For analyzing the colonizing ability of distinct subpopulations, the tumor cells from spontaneous sarcoma and lymphoma of *p53*^−*/*−^ mice were obtained by mechanical disruption. 1 × 10^6^ cells were injected into nude mice through tail vein. The mice were sacrificed to harvest lung, liver, kidney and ovary for histological analysis 3 weeks after injection. The Stellar^high^ and Stellar^low^ (5 × 10^5^) sorted from two independent thymic lymphomas of *p53*^−*/*−^ mice were injected into nude mice through the tail vein. The mice were sacrificed to harvest lung, liver, kidney and ovary for histological analysis 3 weeks after injection. For analyzing the colonization of breast cancer cells to hepatic tissues, 4T1 (1 × 10^6^ cells) were injected into BALB/c mice through the tail vein, and the mice were sacrificed to harvest liver tissues for histology analysis 6 days after the injection. To analyzing the colonization to liver, 4T1 or 168FARN (5 × 10^5^ cells) were injected into BALB/c mice through the tail vein. 4T1 cells (1 × 10^6^ cells) with control or knockout in diverse PGC-related genes were injected into BALB/c mice through the tail vein injection. Two independent knockout clones were used in these experiments. Five mice for each group were used. The mice were sacrificed to harvest liver tissues for histological analysis 15 days after injection.

### Drug treatment for in vivo liver metastasis

4T1 cells (1 × 10^6^ cells) treated with DMSO or DMH2 (6 μM) for 3 days were inoculated into BALB/c mice through the tail vein injection (5 mice for each group). The mice were sacrificed to harvest liver tissues for histological analysis 6 days after injection.

### Isolation of thymic tumor cells

The thymic tumor cells were isolated from thymic tumor obtained from *p53*^−*/*−^ mice by mechanical disruption using two cover slides. Cells were then filtered and used in diverse assays.

### Histology and immunohistochemistry

For histology analysis, the tumors and tissues were fixed in 10% formalin, embedded in paraffin, sectioned and then performed hematoxylin and eosin (H&E) staining or immunohistochemistry staining by standard procedures^33^. The sectioned slides were antigen retrieved (0.01 M Citrate buffer, pH 6.0), incubated with the primary antibody for overnight at 4 °C and then detected by VECTASTAIN Universal Quick HRP Kit (Peroxidase) (Vector Laboratory) with DAB kits (Abcam). Hematoxylin staining was used to show nuclear details.

### Antibodies

The following primary antibodies were used in the study including anti-CXCR4 (ab124824, AbCam), anti-TRAIL (AF1121, R&D), anti-c-Kit (AF1356, Novus Biological), anti-CD47 (ab175388, Abcam), anti-Fasl (ab15285, Abcam), anti-PDL1 (NBP1-76769, Novus Biological), anti-CD4 (ab183685, Abcam), anti-SSEA1 (ab16285, Abcam), anti-Oct4 (ab184665, Abcam), anti-Sox2 (MAB2018R-100, R&D), anti-Nanos3 (ab70001, Abcam), anti-Ifitm3 (ab109429, Abcam), anti-Stellar (Invitrogen, PA5-34601), anti-PRDM14 (ab187881, Abcam), anti-Vasa (ab27591, Abcam), anti-DAZL (NB100-2437, Novus biologicals), anti-SMAD2 (ab33875, Abcam).

### Alkaline phosphatase staining

Cultured cells were fixed with 4% paraformaldehyde in PBS for 4 min, washed twice with a Tris-HCl (pH = 8.2) buffer solution and then stained with AP kit (Vector Laboratory) overnight at room temperature.

### FCS sorting for Stellar^high^ cells

The thymic lymphoma cells were obtained by mechanical disruption with two cover slides from *p53*^−*/*−^ spontaneous thymic lymphoma, stained with the antibody against Stellar for 1 h on ice, stained with goat anti-mouse (Alexa Fluor 488), and subjected to the flow sorter for sorting Stellar^high^ cells.

### Immunofluorescence assay

The cultured cells in chamber slides were fixed in 4% paraformaldehyde in PBS for 20 min, blocked in PBS containing 2.5% BSA and 0.05% Triton-X-100 and then incubated with primary antibody at 4 °C for overnight. Cells were washed, stained with fluorescent dye-conjugated secondary antibodies (Alexa Fluor 488 or 555), followed by 4,6-diamidino-2-phenylindole (DAPI; Invitrogen) staining. The fluorescent images were obtained using the fluorescent microscope.

### Quantification of PGC-like cells in liver

Hepatic tissues were stained with the primary antibody against Oct4, Nanog or Nanos3, and PGC-like cells were counted if cells display positive signals for all these germ cell markers. The ratio of PGC-like cells versus total cells in the cell clusters in liver was measured.

### CRISPR–Cas9 knockout

The CRISPR/Cas9 and mouse sgRNA-RFP plasmids were obtained from Addgene (Plasmid #52961) and Sigma, respectively. The guide empty vector was used to serve as the control (Sigma). Twenty microliters of X-tremeGENE9 DNA transection Reagent (Roche) were mixed with 500 μl Opti-MEM medium (Gibco) for 5 min, incubated with 6 ng of DNA (CRISPR/Cas9, 090, 091 plasmids) for 25 min at room temperature, and these mixtures were added to HEK293T cells for 24 h. The medium with the viruses was harvested and filtrated through a 0.45 µm Steri-Flip filter (Millipore). The collected medium was mixed with 20% fresh culture medium and added to 4T1 cells along with 7 μg/ml polybrene for 4 h and replaced with normal 10% fresh medium. Two days after infection, 4T1 cells were selected with 4 μg/ml blasticidin or 5 μg/ml puromycin for 5 days. Multiple clones derived from single cell cultured in 96-well plates were isolated. Confluent cells were then plated in a 24-well plate and knockout cell clones were selected by sequencing. The sequencing data from knockout cells were shown in Supplementary Table S1.

### Real-time PCR analysis

The RNA extracted from cultured cells mixed with Trizol reagent (Invitrogen) was converted to the cDNA by performing the reverse transcription using a reverse transcription kit (Invitrogen). The cDNA was used to perform real-time PCR analysis using SYBR Green PCR Master Mix Kit (Applied Biosystems) according to our previous study^9^.

### Statistical analysis

All experiments were repeated at least three times. The data were calculated by a student’s *t*-test. A *P*-value with <0.05 was considered statistically significant.

## Supplementary information

Supplemetary information

## Data Availability

All data are available in the main text or the supplementary materials.

## Supplementary information

**Supplementary Information** accompanies the paper at (10.1038/s41421-020-0145-3).
