# Supplementary material for: Identification of primordial germ cell-like cells as liver metastasis initiating cells in mouse tumour models
Source: Cell Discov. 2020 Mar 24;6:15. doi: 10.1038/s41421-020-0145-3 (PMC7090051; doi:10.1038/s41421-020-0145-3)
Supplement: Supplementary file 1 — Supplemetary information [file 41421_2020_145_MOESM1_ESM.pdf]

Supplementary Information

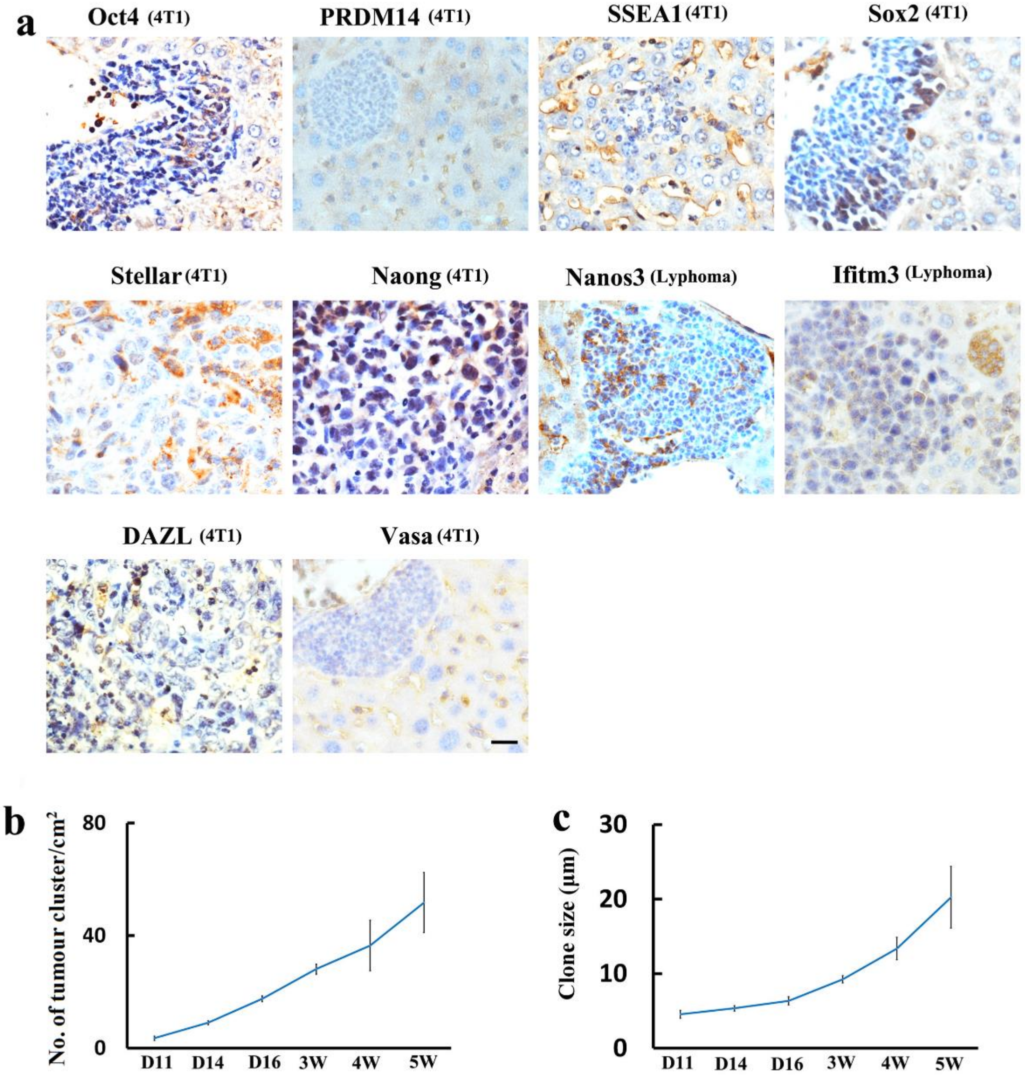

Supplementary Fig. S1. Traits of liver metastasis. (**a**) The differentiated somatic tumour cells in liver of the mice inoculated with lymphoma or 4T1 cells showed low level of the expression of PGC-related markers. (**b**) The plot shows the density of 4T1tumour cells in liver at different time points. (**c**) The plot shows the changes of 4T1 tumour clone size in liver metastases at different time points. Scale bar=50μm.

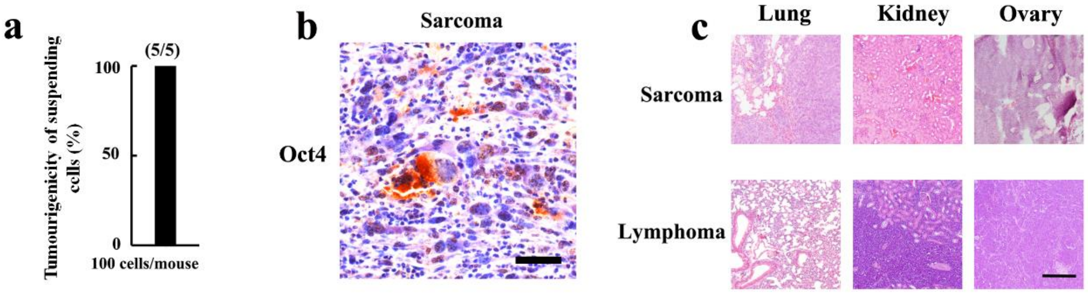

Supplementary Fig. S2. PGC-like cells colonize to liver. (**a**) The plot shows the tumourigenicity of suspended 4T1 cells (100 cells/mouse) subcutaneously injected into nude mice. (**b**) Sarcoma tumour sections from *p53*$^{-/-}$ mice were stained with Oct4 antibody. (**c**) Histological analysis of various tissues from the mice inoculated with sarcomas or lymphomas tumour cells from *p53*$^{-/-}$ mice 15 days after the tail vein injection. **p<0.01. Scale bar= 100 μm (**b**), 200 μm (**c**).

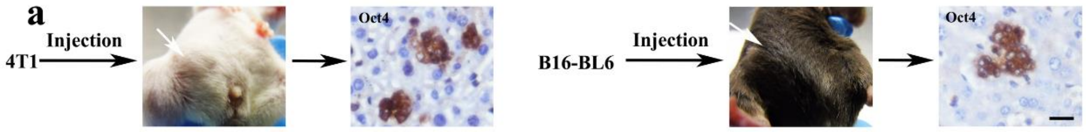

Supplementary Fig. S3. The ability of PGC-like tumour cells to overcome the physical and biological hurdles. (**a**) PGC-like tumour cells survived in mouse liver with normal immune system. Scale bar= 50 μm.

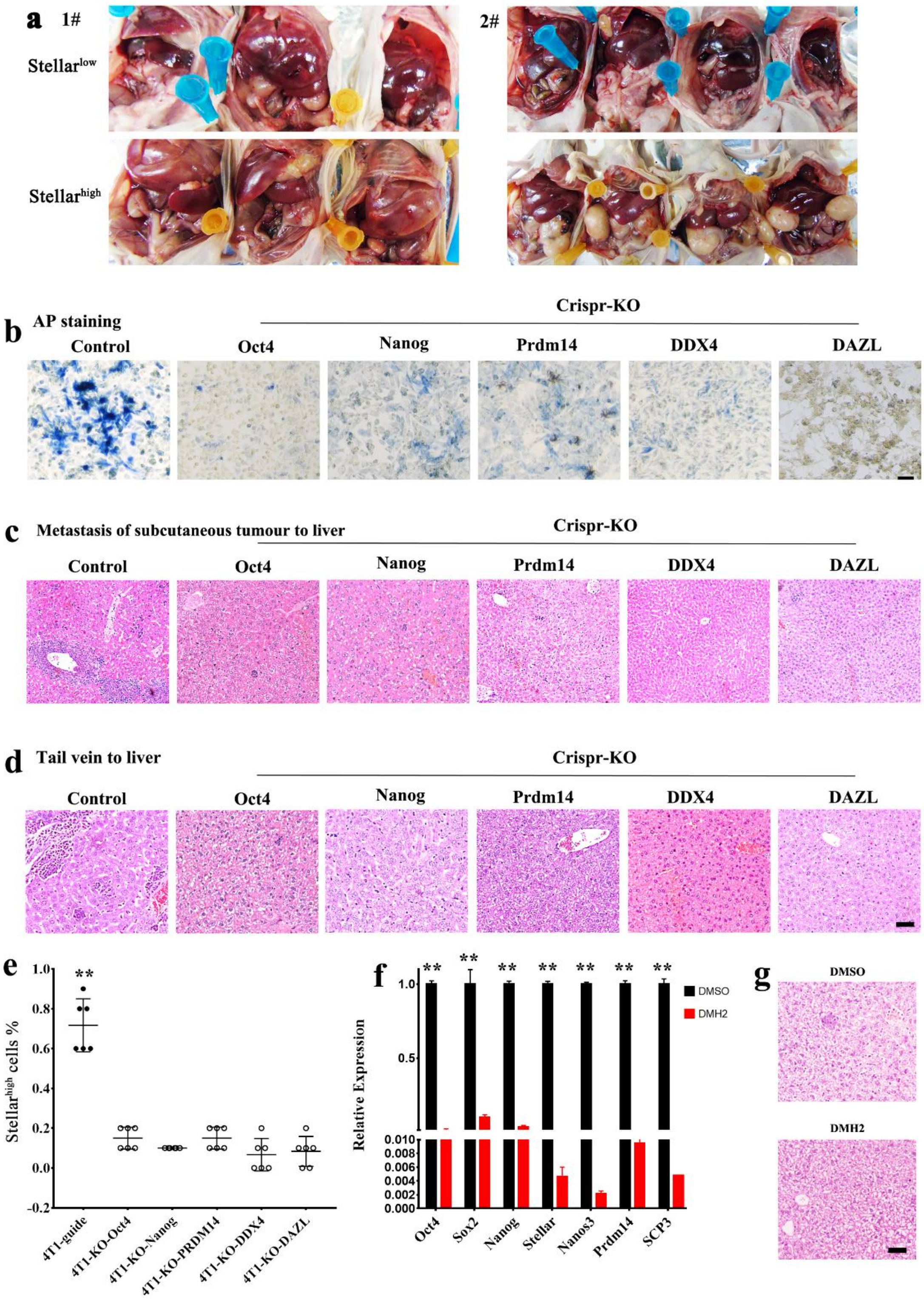

Supplementary Fig. S4. Genetic deletion of the genes critical for PGC development represses the formation of PGC-like cells and liver metastasis. (**a**) The images of various tissues from the mice inoculated with $\text{Stellar}^{\text{low}}$ and $\text{Stellar}^{\text{high}}$ tumour cells sorted from two different lymphomas of $p53^{-/-}$ mice through the tail vein injection (arrow showed the ovary tumours). (**b**) AP staining of 4T1 cells with control and

knockout in diverse PGC-related genes. (**c**) Histological analysis of liver tissues from the mice subcutaneously injected with 4T1 cells with control and knockout in diverse PGC-related genes 25 days after injection. (**d**) Histological analysis of liver tissues from the mice inoculated with 4T1 cells with control and knockout in diverse PGC-related genes 15 days after the tail vein injection. (**e**) Stellar^{high} tumour cells were determined by FCS in 4T1 cells with control and knockout in diverse PGC-related genes. (**f**) The real-time PCR analysis of the expression of PGC-related genes in 4T1 cells treated with DMSO or DMH2 (6 μM) for 3 days. (**g**) Histological analysis of the liver tissues of the mice injected with 4T1 cells treated with DMSO or DMH2 (6 μM) for 3 days after 6 days of the tail vein injection. **$p<0.01$. Scale bar= 50 μm.

Supplementary Table S1. Sequencing data of CRIPSR-Cas9 knockout 4T1 cells.

<table>
<tr><th>Gene</th><th>Knock out DAN with CRISPR-Cas9</th></tr>
<tr><td>Oct4-1#</td><td>Query 74 GAGGATCACCTTGGGGTACACCCAGGCCGAC----CGCGCCGCCCGGGCTGTTTGTTTGG 129
||||||||||||||||||||||||||||||| || | || |||| | | |||||
Sbjct 35509165 GAGGATCACCTTGGGGTACACCCAGGCCGACGTGGGGCTCACCCTGGGCGTTCTCTTTGG 35509224
sgRNA: GTACACCCAGGCCGACGTGGGG Del:4bp</td></tr>
<tr><td>Oct4-2#</td><td>Query 62 CTGAAGCAGAAGAGGATCACCTTGGGGTACACCCAGGCCGACCGTGGGGCTCACCCTGGG 121
|||||||||||||||||||||||||||||||||||||||||| |||||||||||||||||
Sbjct 35509154 CTGAAGCAGAAGAGGATCACCTTGGGGTACACCCAGGCCGAC-GTGGGGCTCACCCTGGG 35509212
sgRNA:GTACACCCAGGCCGACGTGGGG Int:1bp</td></tr>
<tr><td>Nanog-1#</td><td>Query 240 AAGCAGAAGATGCGGACTGTGTTCTCACACGCCCAGCTGTGTGCCCTC------------ 287
|||||||||||||||||||||||||| || |||||||||||||| |||
Sbjct 122711672 AAGCAGAAGATGCGGACTGTGTTCTCTCAGGCCCAGCTGTGTGCACTCAAGGACAGGTTT 122711731
sgRNA: GCTGTGTGCACTCAAGGACAGG Del:37bp
Query 288 ----AGCAG---------AGGCTCC------------AAATCTCATCCATTCCGACCCCG 322
||||| || |||| || |||| ||||||| || || |
Sbjct 122711732
CAGAAGCAGAAGTACCTCAGCCTCCAGCAGATGCAAGAACTCTCCTCCATTCTGAACCTG 122711791</td></tr>
<tr><td>Nanog-2#</td><td>Query 244 AAGATGC 250
|||||||
Sbjct 122711678 AAGATGC 122711684 Del:86bp
sgRNA: GCTGTGTGCACTCAAGGACAGG
Query 1 AAGAACTCTCCTCCATTCTGAACCTGAGCTATAAGCAGGTGGGGTCCTTAAAAGTTGCT- 59
|||||||||||||||||||||||||||||||||||||||||||||||||| ||||||||
Sbjct 122711766 AAGAACTCTCCTCCATTCTGAACCTGAGCTATAAGCAGGTGGGGTCCTTAGAAGTTGCTG 122711825</td></tr>
<tr><td>Prdm14-1#</td><td>Query 61 GTAGAGTGGTCAACGCCCGCGATGTAGAGGAAGTACAGTGTACTATCTCTCGGTTGGTGG 120
||||||||||||| ||| |||| || ||| | |||| | || | ||||||| | ||||
Sbjct 13124421 GTAGAGTGGTCAATGCCAGCGAAGTGAAGGCA-CACAG-GGAC-AACTCTCGGAT-GTGG
sgRNA: GTCAATGCCAGCGAAGTGAAGG Int: 4bp</td></tr>
<tr><td>Prdm14-2#</td><td>Query 662 TGGACCCTTTCGAG 675
||||||||||||||
Sbjct 13124435 TGGACCCTTTCGAG 13124422 Del: 323bp
sgRNA: GTCAATGCCAGCGAAGTGAAGG
Query 1 GCATGCTACAGGAAAGAAAGACAAGTTTTAGACTTTCATGTTACTT 46</td></tr>
</table>

<table>
<tr><td></td><td>||||||||||||||||||||||||||||||||||||||||||||||
Sbjct 13124098 GCATGCTACAGGAAAGAAAGACAAGTTTTAGACTTTCATGTTACTT 13124053</td></tr>
<tr><td>DDX4-1#</td><td>Query 243 ATTTTTCAGATATTGGCGAGTCTAGTAAAAAAGAGAACACATCTAC--GTGGGGGTTT 300
|||||||||||||||||||||||||||||||||||||||||||||| ||| || |||
Sbjct 112636055 ATTTTTCAGATATTGGCGAGTCTAGTAAAAAAGAGAACACATCTACAACTGGTGGCTTT 112635996
sgRNA: AGAACACATCTACAACTGGTGG Del: 2bp</td></tr>
<tr><td>DDX4-2#</td><td>Query 251 ATATTGGCGAGTCTAGTAAAAAAGAGAACACATCTACAACT--------TGGAAGAGGAA 302
||||||||||||||||||||||||||||||||||||||||| |||||||||||
Sbjct 112636045 ATATTGGCGAGTCTAGTAAAAAAGAGAACACATCTACAACTGGTGGCTTTGGAAGAGGAA 112635986
sgRNA: AGAACACATCTACAACTGGTGG Del: 8bp</td></tr>
<tr><td>DAZL-1#</td><td>Query 309 TCATGCCAAACACCGTTTTTGT----AGAATAGATGTATGTCAATCGAATTTATATTTAA 364
|||||||||||||||||||||| |||| ||||| | || | ||| |||||| |
Sbjct 50289132 TCATGCCAAACACCGTTTTTGTTGGAGGAATTGATGTTAGGGTATTGTATTCATATTTCA 50289073
sgRNA: CAAACACCGTTTTTGTTGGAGG Del: 4bp</td></tr>
<tr><td>DAZL-2#</td><td>Query 310 ATGCCAAACACCGTTTTTG-TGGAGGAAATGATGTTAGGGTATTGTATTCATATTTCATT 368
||||||||||||||||||| |||||||| |||||||||||||||||||||||||||||||
Sbjct 50289130 ATGCCAAACACCGTTTTTGTTGGAGGAATTGATGTTAGGGTATTGTATTCATATTTCATT 50289071
sgRNA: CAAACACCGTTTTTGTTGGAGG Del: 1bp</td></tr>
<tr><td>SMAD2-1#</td><td>Query 302 TTAAAAGTGCTCTCCTTTTTTCCCTTGCTTCCAAATAGGTCTCTTGACGGCC-------- 353
|||||||||||||||||||||||||||||||||||||||||||||| ||||
Sbjct 76287890 TTAAAAGTGCTCTCCTTTTTTCCCTTGCTTCCAAATAGGTCTCTTGATGGCCGTCTTCAG 76287949
sgRNA: TCTCTTGATGGCCGTCTTCAGG Del:19bp
Query 354 -----------GAAAGGGTTGCCACATGTTATATATTGCCGGCTCTGGCGCTGGCCGGAC 402
|||||||||||||||||||||||||||||||||||||||||||||||||
Sbjct 76287950 GTTTCACACCGGAAAGGGTTGCCACATGTTATATATTGCCGGCTCTGGCGCTGGCCGGAC 76288009</td></tr>
<tr><td>SMAD2-2#</td><td>Query 491 AAGATGAAGTGTGTGTAAATCCGTACCACTA-CAGAGAGTTGACACCCCAGGTAAGAAAC 549
||||||||||||||||||||||||||||||| ||||||||||| |||||||||| |||||
Sbjct 76288071 AAGATGAAGTGTGTGTAAATCCGTACCACTACCAGAGAGTTGAGACCCCAGGTAGGAAAC 76288130
sgRNA: CCACTACCAGAGAGTTGAGACC Del:1bp</td></tr>
</table>
